# The combination of FLCWK with 5‐FU inhibits colon cancer and multidrug resistance by activating PXR to suppress the IL‐6/STAT3 pathway

**DOI:** 10.1111/jcmm.70185

**Published:** 2024-11-04

**Authors:** Lifan Zhong, Qianru Wang, Zhixiong Kou, Lianfang Gan, Zhaoxin Yang, Junhua Pan, Ling Huang, Yunqiang Chen

**Affiliations:** ^1^ School of Hainan Provincial Drug Safety Evaluation Research Center Hainan Medical University Haikou China; ^2^ Department of Key Specialist Office Sanya Hospital of Traditional Chinese Medicine Sanya China; ^3^ Center for Pharmacovigilance of Hainan Province Hainan Medical Products Administration Haikou China; ^4^ Department of Rehabilitation therapeutics The Second Affiliated Hospital of Hainan Medical University Haikou China

**Keywords:** 5‐fluorouracil, Colon cancer, Fengliao changweikang, multidrug resistance, Pregnane X Receptor

## Abstract

5‐fluorouracil (5‐FU) is a preferred chemotherapeutic agent for the treatment of colon cancer. Nonetheless, its clinical effectiveness is frequently hampered by suboptimal therapeutic outcomes and the emergence of drug resistance. Therefore, there exists a pressing demand for novel therapeutic agents to circumvent chemoresistance. The pregnane X receptor (PXR) exerts a pivotal regulatory influence on the proliferation, invasion, and chemoresistance mechanisms in colon cancer. Activation of PXR drives up the transcription of the multidrug resistance gene (MDR1), thus prompting the expression of P‐glycoprotein (P‐gp) responsible for conferring tumour resistance. This study scrutinized the potential of Fengliao Changweikang (FLCWK) in augmenting the efficacy of 5‐FU in the management of colon cancer. To this end, we engineered colon cancer cells with varied levels of PXR expression via lentiviral transfection, subsequently validating the findings in nude mice. By means of MTT assays, flow cytometry apoptosis analysis, Western blotting and immunofluorescence, we probed into the prospective impacts of FLCWK and 5‐FU on cellular viability and resistance. Our results revealed that while upregulation of PXR amplified the therapeutic benefits in colon cancer treatment, it concurrently heightened resistance levels. FLCWK demonstrated a capacity to reduce P‐gp expression, with the combined administration of FLCWK and 5‐FU effectively reversing resistance mechanisms. Furthermore, activation of PXR was found to impede the IL‐6/STAT3 signalling pathway. In an effort to mimic the development of colon cancer, we established an azomethane oxide (AOM)/ dextran sodium sulfate (DSS) mouse model, showing that FLCWK bolstered the inhibitory effects of 5‐FU, impeding the progression of colon cancer. In summation, our findings point towards the potential of FLCWK in the treatment of colon cancer, particularly in strengthening the therapeutic efficacy of 5‐FU in the prevention and control of the disease.

## 1 INTRODUCTION

Colon cancer remains the third most prevalent cancer globally. According to the latest 2023 cancer statistics, it ranks third both in incidence and mortality.^1^ The predominant treatment approach for most colon cancer patients involves chemotherapy followed by surgical intervention. Despite advancements in targeted therapies that have enhanced chemotherapy protocols, only a minority of patients exhibit a favourable response, while the majority tend to develop resistance.^2^, ^3^ Consequently, it is imperative to delve into the molecular mechanisms at the core of colon cancer and uncover novel treatment modalities to bolster the efficacy of existing chemotherapy agents and diminish the emergence of resistance.

The Pregnane X Receptor (PXR) is an orphan nuclear receptor that wields significant regulatory control over the growth,^4^ invasion and chemotherapy resistance in colon cancer through its activation by both endogenous and exogenous ligands.^5^, ^6^ Activation of PXR triggers the transcription of multidrug resistance gene 1 (MDR1), culminating in the expression of P‐glycoprotein (P‐gp), a key player in mediating tumour resistance.^7^ Additionally, PXR showcases the ability to modulate the proliferation and apoptosis dynamics of colon cancer cells and thwart chronic colitis‐associated cancer (CAC) cell proliferation by engaging with cytokines such as IL‐6.^8^ Research indicates that PXR activation can reduce the signal transducer and activator of transcription 3 (STAT3) phosphorylation levels, dampen the production of inflammatory cytokines and contribute positively to upholding the integrity of the intestinal epithelial barrier.^9^, ^10^

5‐ fluorouracil (5‐FU) is a fundamental chemotherapeutic agent utilized in the pre‐ and post‐operative settings as well as for palliative care across various stages of colon cancer.^11^, ^12^ The emergence of multidrug resistance (MDR) constitutes a significant factor contributing to chemotherapy failures,^13^ with a notable proportion of colorectal cancer patients displaying an abnormal increase in MDR levels, leading to a substantial decrease in the effectiveness of diverse drugs. It has been shown that stable inhibition of MDR1 can reverse drug‐resistant phenotype of CRC cells.^14^ However, in order to become a successful modality of therapy, further improvements are needed in the design of this inhibitors. Research has shown that MDR1 is one of the downstream genes of TCF/LEF transcriptional complex. Therefore, silencing LEF1 can affect the expression of the MDR1 and make the cells more sensitive to the chemotherapeutic drugs.^15^ miRNAs play roles in resistance to anti‐cancer drugs by modulating different signalling pathways. Related studies have shown that a subset of miRNAs may be associated with 5‐Fluorouracil sensitivities by targeting Tyms and Abcg2 genes which are involved in drug resistance.^16^

Particularly, the prevalent form of MDR is closely associated with the overexpression of P‐gp,^17^, ^18^, ^19^ which impedes the accumulation of 5‐FU within cells, presenting a critical hurdle in cancer treatment involving this drug. Despite the development of several inhibitors targeting P‐gp, challenges such as the significant toxicity of these inhibitors or complex factors related to the pharmacokinetics and distribution of anticancer medications have impeded the achievement of favourable outcomes in clinical trials.^20^ The issue of drug resistance in anticancer therapy persists as a major impediment in the field of cancer treatment.^11^ Therefore, strategies focused on enhancing the sensitivity of tumour cells to chemotherapy agents and reversing MDR through the synergistic use of medications have emerged as pivotal approaches to elevate the overall efficacy of chemotherapy.^21^, ^22^

In comparison to the combination of conventional chemical drugs, traditional Chinese medicine offers advantages such as lower toxicity and the ability to target multiple sites, effectively reversing various mechanisms of tumour resistance. Fengliao Changweikang (FLCWK) is recognized as a second‐class protected Chinese patent medicine in China and is extensively used in clinical practice to address acute and chronic gastrointestinal inflammation, irritable bowel syndrome, ulcerative colitis and other gastrointestinal disorders. Noteworthy is its substantial clinical efficacy devoid of adverse reactions or toxic side effects, ensuring a high level of safety.^23^, ^24^ Previous investigations from our research team demonstrated that the combination of FLCWK with 5‐FU suppressed the proliferation and induced apoptosis in colon cancer cells by modulating the IL‐6/STAT3 signalling pathway, resulting in the inhibition of colon tumour growth in CAC mice.^25^, ^26^, ^27^

In this study, our objective was to assess the impact of combining FLCWK with 5‐FU on colon cancer development in a mouse model of CAC and a xenograft model of human colon cancer cells, as well as investigate the effect of FLCWK on resistance to 5‐FU. Building on these findings, we also explored the influence of varying PXR expressions on colon cancer cell lines. Our results indicated that PXR activation led to increased proliferation and MDR in colon cancer cells; however, the concurrent administration of FLCWK with 5‐FU effectively reversed the MDR linked to 5‐FU, offering significant implications for future clinical applications.

## 2 MATERIALS AND METHODS

### 2.1 Ethics statement

This study was ethically approved by the Ethics Committee of Hainan Medical University under Animal Ethics No: HYLL‐2021‐086.

### 2.2 Drugs and reagents

Fluorouracil injection was sourced from Xudong Haipu Pharmaceutical Co., Ltd. (Shanghai, China). FLCWK granules were obtained from Haikou Pharmaceutical Co. Ltd. (Haikou, China). MTT was procured from Sigma (St. Louis, MO, USA). The Annexin V‐FITC Detection Kit was purchased from Beyotime Biotechnology Co. Ltd. (Shanghai, China). PVDF membranes were sourced from Millipore (Billerica, MA, USA). Goat anti‐rabbit and anti‐mouse secondary antibodies were supplied by Boster Biological Technology Co., Ltd. (Wuhan, China). Azomethane oxide (AOM) was obtained from Sigma (St. Louis, MO, USA). Dextran sodium sulfate (DSS) was procured from MP Biomedicals (Santa Ana, CA, USA). The lentiviral transfection kit was purchased from Shanghai GeneChem Co., Ltd.

### 2.3 Cell culture

Colon cancer Lovo cells were procured from Pricella Biotechnology Co., Ltd. (Wuhan, China) and underwent characterization using short tandem repeats (STR) analysis. Additionally, they were examined under an electron microscope to confirm the absence of mycoplasma contamination. The Lovo cells were cultured in 1640 medium supplemented with 10% fetal bovine serum at 37°C in a 5% CO_2_ humidified atmosphere incubator. Cell passaging was conducted every 2–3 days by trypsinization at a concentration of 0.25%. For all experiments, cells in the logarithmic growth phase exhibiting good viability were utilized.

### 2.4 Cell transfection and lentivirus infection

The lentiviruses expressing GV358‐LV‐NR1I2 as well as the GV493‐NR1I2‐RNAi were obtained from Gene (Shanghai Genechem Co., Ltd.). Lovo cells were seeded in 12‐well plates with RPMI 1640 medium supplemented with 10% fetal bovine serum to reach 50% confluence by the following day. The indicated cells were transduced with the corresponding lentiviral vectors (MOI: 40). After 72 h, cells were screened using puromycin dihydrochloride. The efficiency of lentiviral transfection was assessed through Real‐time PCR and Western blot analysis.

### 2.5 Cell viability assay

About 1 × 10^4^ of each cell line were seeded in 96‐well plates and allowed to adhere for 1h. Subsequently, the supernatant was removed, and various treatment groups were introduced for a 48‐h incubation period. Cell viability was assessed using the MTT kit, and the absorbance was measured at 450 nm utilizing a microplate reader.

### 2.6 Cell apoptosis assay

About 1 × 10^5^ of each cell line were seeded in 12‐well plates. Following a 24‐h incubation period with drug treatment, apoptosis levels in Lovo cells were evaluated using the Annexin V fluorescein isothiocyanate (FITC)/propidium iodide (PI) apoptosis detection kit (Beyotime Biotechnology Co. Ltd., Shanghai, China). The analysis was performed using flow cytometry.

### 2.7 Subcutaneous xenograft model

Four‐week‐old male BALB/c nude mice obtained from the Institute of Model Animals at Nanjing University were housed in a specific pathogen‐free (SPF) facility. Following a week of acclimatization, the mice were inoculated with Lovo cells, GV358‐LV cells, and GV493‐RNAi cells. Each cell type was categorized into four groups: model group, FLCWK group, 5‐FU group and FLCWK+5‐FU group, comprising a total of 12 groups with 8 mice in each group. The Lovo cells, GV358‐LV cells, and GV493‐RNAi cells were subcutaneously injected at a cell concentration of 1.0 × 10^7^ cells/mL, with each mouse receiving a 100 μL injection. Commencing from the day of cell injection, mice in the FLCWK group and the FLCWK+5‐FU group were orally administered FLCWK (4 g/kg) on the day of inoculation, and this administration was continued once daily until the conclusion of the experiment. Once the tumours reached approximately 100 mm^3^ in size, tail vein administration of 5‐FU (24 mg/kg) was initiated in mice in the 5‐FU group and FLCWK+5‐FU groups once every 3 days for a total of seven administrations.^26^, ^27^ Tumour measurements were recorded every 2 days starting from the day of inoculation. Tumour volume was calculated using the formula *V* = ab^2^/2, where ‘a’ and ‘b’ represent the length and width of the tumour, respectively. Upon completion of the modelling, the mice were humanely euthanized, and tumour tissues were extracted, photographed for size observation and weighed. Subsequently, a portion of the tumour tissue was fixed in 4% neutral formaldehyde, while the remaining tissue was stored at −80°C for subsequent gene and protein detection tests.

### 2.8 Induction of colitis and CAC

Four‐week‐old male BALB/c mice acquired from Hunan SJA Laboratory Animal Co., Ltd. were housed in SPF environments, and the CAC model was induced by intraperitoneal injection of AOM (12.5 mg/kg) followed by 3 cycles of 2.5% DSS treatment, as previously described. Prophylactic administration of FLCWK (4 g/kg) was initiated after a single intraperitoneal injection of AOM on the same day, continuing once daily until the end of the modelling. The mice underwent 3 cycles, each consisting of 1 week of 2.5% DSS in drinking water followed by 2 weeks of regular water. At the completion of the 3 cycles, tail vein injections of 5‐FU (24 mg/kg) began 1 week before the modelling endpoint and were administered daily for seven consecutive days^26^, ^27^ (Figure 5A). On day 70, all mice were euthanized, and their colons were extracted. The number of tumours was counted, and the colons were incised longitudinally to observe tumour lesions on the intestinal lining after washing out faeces with saline. A section of the colon tissue was fixed in 4% neutral formaldehyde, while the remaining tissue was stored at −80°C for subsequent gene and protein detection tests.

### 2.9 Histological analysis

Sections of the subcutaneous grafted tumour and colon tissue from the mice were fixed as previously outlined. The tissues underwent a process of gradient dehydration, paraffin embedding, sectioning and HE staining, after which they were examined for histopathological alterations under a light microscope.

### 2.10 Immunohistochemistry and immunofluorescence

Colonic pathology was assessed utilizing a previously established histological scoring system. Paraffin sections of colon tissues from CAC mice and tumour tissues from the genetically identical mice were dewaxed and subjected to antigen retrieval. The sections were then treated with specific primary antibodies overnight at 4°C under light‐protected conditions. For immunohistochemistry, sections were subsequently exposed to mouse ki‐67 antibody (1:200) overnight, followed by incubation with horseradish peroxidase‐conjugated goat anti‐mouse secondary antibody (1:1000) and haematoxylin staining. In the case of immunofluorescence, sections were incubated with primary antibodies, including P‐STAT3 (1:100), PXR (1:100) and P‐gp (1:100) overnight at 4°C. This was followed by incubation with fluorescently labelled secondary antibodies (1:300) for 1 h at room temperature under light‐protected conditions. Finally, the samples were stained with DAPI and sealed.

### 2.11 Realtime‐PCR

Logarithmically grown in three cell lines were cultured and seeded in six‐well plates, followed by treatment with FLCWK, 5‐FU, or a combination of both. After 48 h, total RNA was extracted from the cells and reversely transcribed to cDNA using the cDNA Reverse Transcription Kit (TaKaRa, Japan). The synthesized cDNA was then amplified using SYBR Green PCR Master Mix (Invitrogen, USA) and analysed with Real‐time PCR on an Applied Biosystems Quant Studio 6 Flex Real‐Time PCR System (Applied Biosystems, Foster City, CA, US). The primers used for amplification were as follows: PXR forward 5′‐CTTGGCAGTGTCCATCTGTCTT‐3′, reverse 5′‐CAGCCTGCTCATAGGTTCTTGTT‐3′; MDR1 forward 5′‐GGGAGCTTAACACCCGACTTA‐3′, reverse 5′‐GCCAAAATCACAAGGGTTAGCTT‐3′; GAPDH forward 5′‐GGAGCGAGATCCCTCCAAAAT‐3′, reverse 5′‐GGCTGTTGTCATACTTCTCATGG‐3′.

### 2.12 Western blot

Cells and tumour tissues were lysed in RIPA buffer (P0013B, Beyotime Biotech, Shanghai, China), and the extracted proteins were separated by SDS‐PAGE (8%–14%) and transferred onto PVDF membranes (Millipore, MA, US). The membranes were then probed with primary antibodies against PXR (1:500), P‐gp (1:1000), STAT3 (1:1000), P‐STAT3 (1:2000) from Cell Signalling Technology (CST), IL‐6 (1:1000), IL‐1β (1:1000), CyclinD1 (1:200), CDK‐4 (1:2000), Bcl‐2 (1:1000), BAX (1:1000) and β‐actin (1:10,000) from Abcam. Subsequently, the membranes were incubated with secondary antibodies, goat anti‐rabbit IgG (1:8000) and goat anti‐mouse IgG (1:8000) from Boster Biological Technology Co., Ltd., and visualized using enhanced chemiluminescence (ECL) reagents from New Cellular and Molecular Biotechnology. The band intensities were quantified using ImageJ software for analysis.

### 2.13 Statistical analysis

All evaluations of data were made using either GraphPad Prism (version 8.0) or SPSS (version 21.0, Abbott Laboratories, USA). Unpaired *t*‐test was used for comparison between groups and multiple comparisons were performed using one‐way analysis of variance (ANOVA). The data were presented as the mean ± SEM. A significance level of *p* < 0.05 was considered statistically significant.

## 3 RESULTS

### 3.1 Successfully constructed overexpression and silencing of PXR cells

**FIGURE 1:**
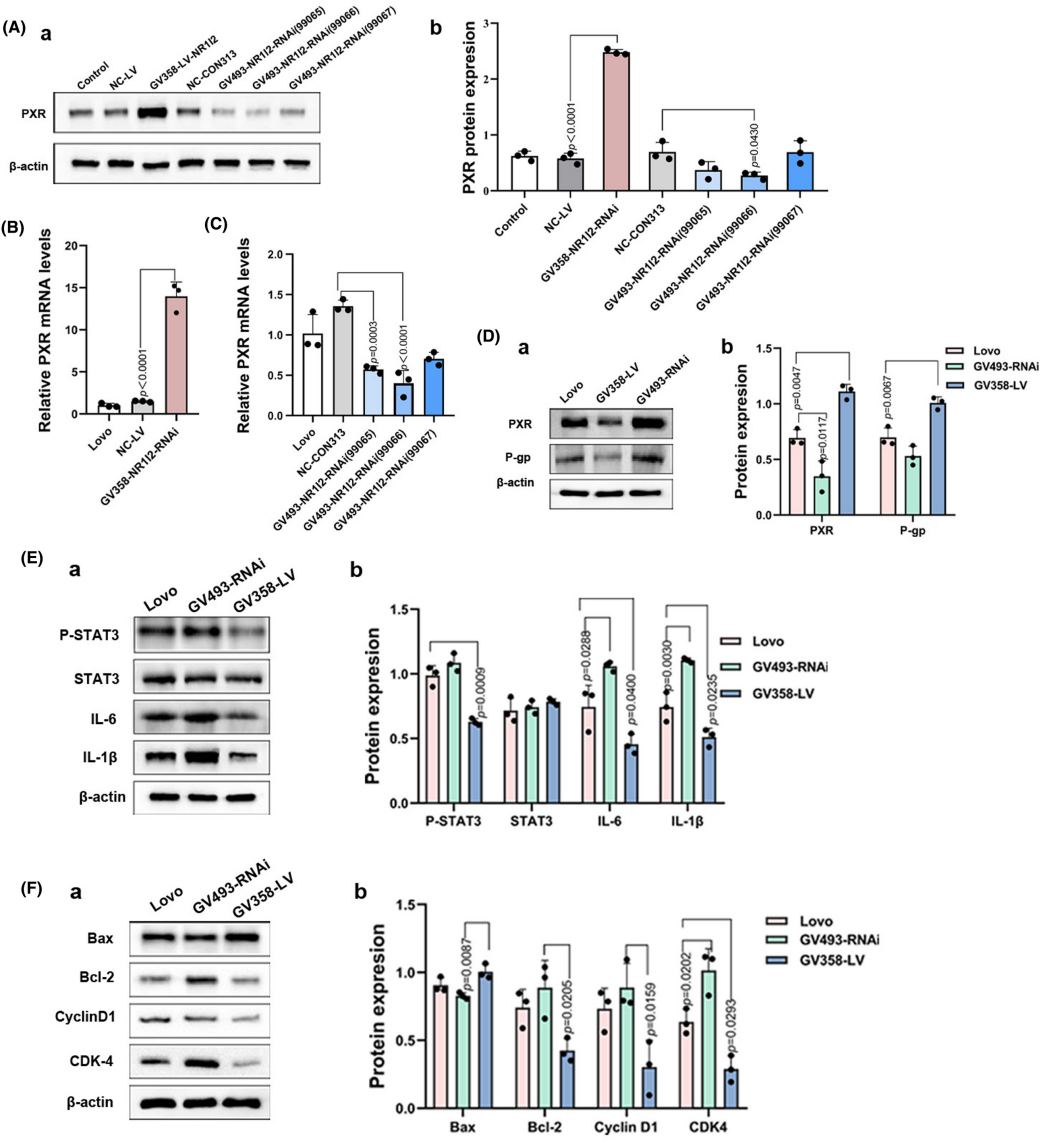
Overexpression of PXR can inhibit IL‐6/STAT3 pathway in Lovo cells. (A) After lentiviral infection, PXR protein was overexpressed in GV358‐LV cells, and silenced in GV493‐RNAi cells by Western blot analysis. (B, C) After lentiviral infection, the mRNA of PXR was overexpressed in GV358‐LV cells, and silenced in GV493‐RNAi cells by Real‐time PCR. (D) The protein levels of PXR and P‐gp in Lovo cells, GV493‐RNAi cells and GV358‐LV cells were assessed by Western blot analysis. (E) The protein levels of P‐SATA3, STAT3, IL‐6, IL‐1β in Lovo cells, GV493‐RNAi cells and GV358‐LV cells were assessed by Western blot analysis. (F) The protein levels of Bax, Bcl‐2, CyclinD1 and CDK‐4 in Lovo cells, GV493‐RNAi cells and GV358‐LV cells were assessed by Western blot analysis.

Previous studies have shown that FLCWK can enhance the effectiveness of 5‐FU in inhibiting proliferation and inducing apoptosis in HT29 and HCT116 colon cancer cells.^26^, ^27^ In this study, we focused on the Lovo cell line to explore the potential role of PXR. We established Lovo cell lines with varying PXR expression levels using lentivirus infection. The results showed that compared with the NC‐LV group, the PXR expression level of GV358‐LV‐NR1I2 was significantly upregulated. And compared with NC‐CON313, the PXR expression level of GV493‐NR1I2 RNAi (99066) was significantly downregulated (Figure 1A–D). The above results indicate that the overexpression and silencing efficiency of PXR in Lovo cells are good and can meet the requirements of subsequent experiments. The cell line with high PXR expression was named GV358‐LV cells, while the cell line with the lowest PXR expression was named GV493‐RNAi cells.

### 3.2 Overexpression of PXR inhibits IL‐6/STAT3 pathway in vitro

To validate the role of PXR in the IL‐6/STAT3 pathway, we conducted Western blot analyses on three cell lines with varying PXR expression levels in vitro. The IL‐6/STAT3 pathway involves key proteins like Bcl‐2 and Bax, crucial for tumour cell apoptosis and Cyclin D1 and CDK‐4, which contribute to tumour cell proliferation. Our in vitro assays revealed a notable decrease in P‐STAT3 levels and reduced expression of IL‐6 and IL‐1β in GV358‐LV cells (Figure 1E). Additionally, in GV358‐LV cells, the protein levels of Bcl‐2, Cyclin D1 and CDK‐4 were downregulated compared to control cells, while Bax expression was upregulated (Figure 1F).

### 3.3 In GV358‐LV cell, FLCWK combined with 5‐FU inhibited cell viability most significantly

**FIGURE 2:**
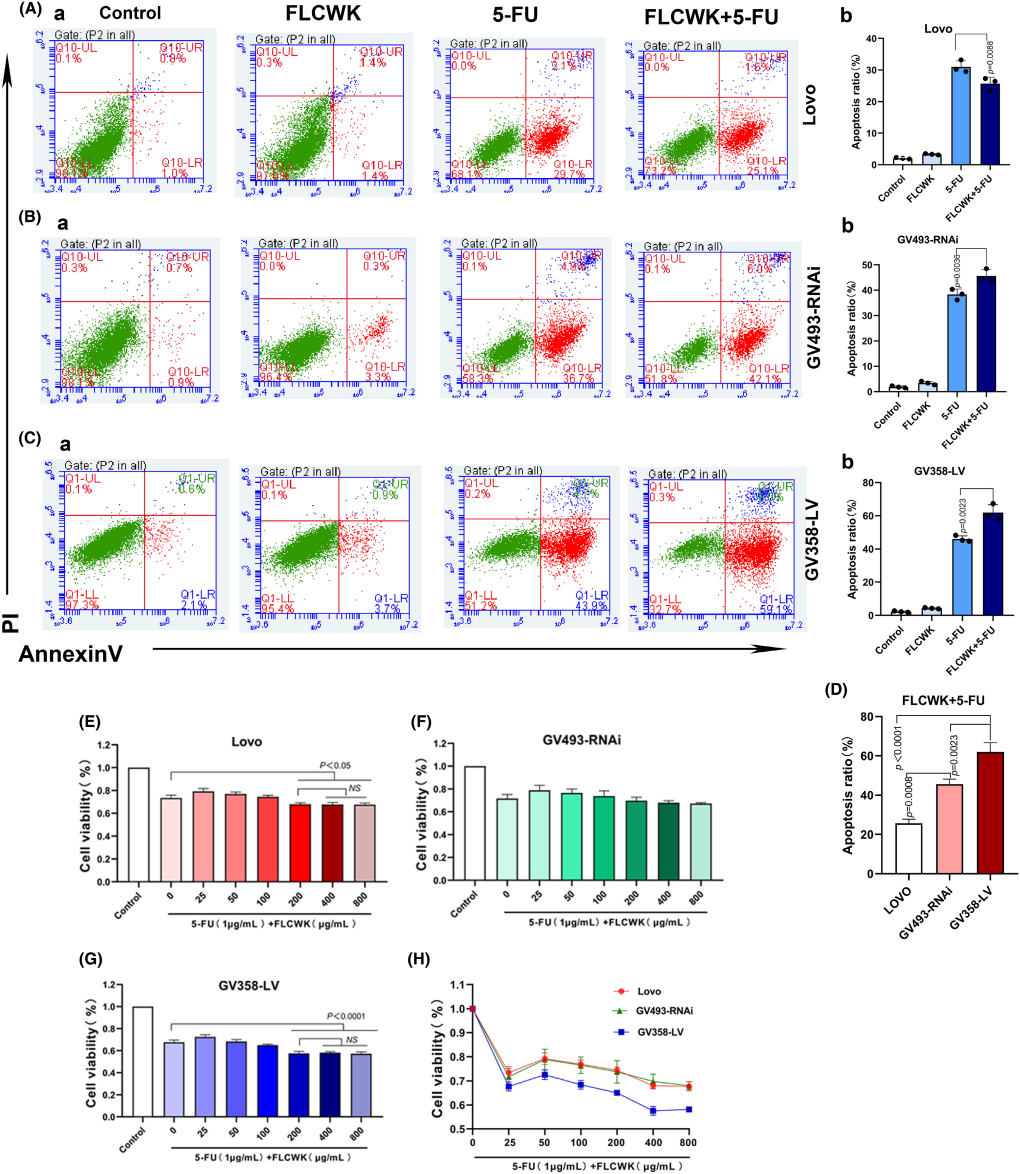
The effects of FLCWK and 5‐FU on the function of Lovo cells with different levels of PXR expression. (A) Detection of apoptosis in Lovo cells by flow cytometry. (B) Detection of apoptosis in GV493‐RNAi cells by flow cytometry. (C) Detection of apoptosis in GV358‐LV cells by flow cytometry. (D) Statistics on apoptosis rate of three cell lines by FLCWK combined with 5‐FU. (E) Cell viability assessed by MTT assay in Lovo cells. (F) Cell viability assessed by MTT assay in GV493‐RNAi cells. (G) Cell viability assessed by MTT assay in GV358‐LV cells. (H) The effect of FLCWK combined with 5‐FU on the viability of three cell lines.

Following treatment with FLCWK and 5‐FU for 48 h, we observed the most significant reduction in cell viability in the GV358‐LV cell line when high‐dose FLCWK was combined with 5‐FU (Figure 2E–H). In contrast, there was no notable difference in cell viability between using 5‐FU alone and its combination with FLCWK in the GV493‐RNAi cells. There was no significant difference in the effect of three doses of FLCWK combined with 5‐FU on cell viability. For subsequent experiments, the concentration of FLCWK in the combination treatment was set at 200 μg/mL.

### 3.4 The apoptotic effect of FLCWK combined with 5‐FU on colon cancer cells

Using flow cytometry to observe the effects of FLCWK and 5‐FU administration on apoptosis in three cell lines. The results showed that there was no significant difference between FLCWK alone and the control group in the three cell lines. In the GV493 RNAi cell line and GV358 LV cell line, the apoptosis rate of the FLCWK combined with 5‐FU group was significantly higher than that of the 5‐FU group compared to the 5‐FU group alone (Figure 2A–C). And it was found that after FLCWK combined with 5‐FU administration, the apoptosis rate of GV358‐LV cell line was significantly higher than that of Lovo cell and GV493 RNAi cell line (Figure 2D). The above results indicate that higher PXR levels were associated with enhanced apoptosis in colon cancer cells with combination therapy.

### 3.5 FLCWK can reverse the 5‐FU resistance induced by PXR activation in vitro

**FIGURE 3:**
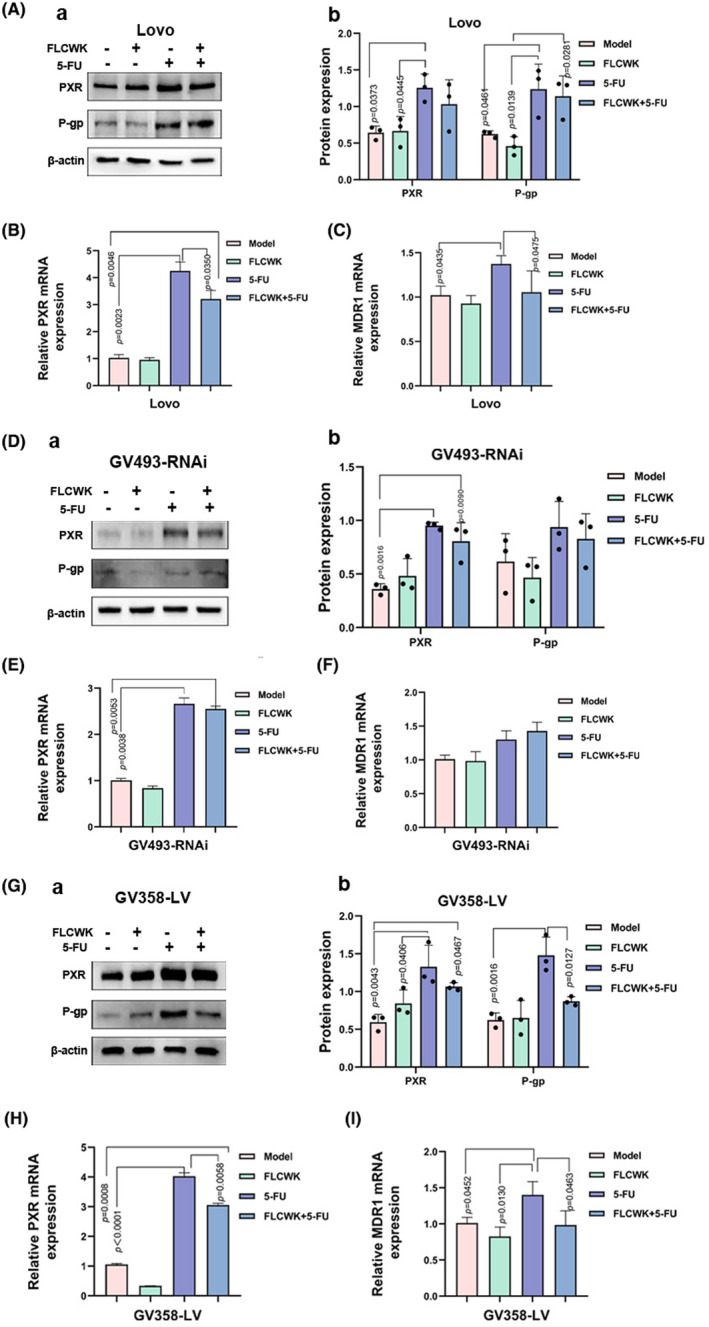
FLCWK can reverse the 5‐FU resistance induced by PXR activation in vitro. (A) The protein levels of PXR, P‐gp, in Lovo cells were assessed by Western blot analysis. (B, C) The mRNA levels of PXR and MDR1 in Lovo cells were quantified by Real‐time PCR. (D) The protein levels of PXR, P‐gp, in GV493‐RNAi cells were assessed by Western blot analysis. (E, F) The mRNA levels of PXR and MDR1 in GV493‐RNAi cells were quantified by Real‐time PCR. (G) The protein levels of PXR, P‐gp, in GV358‐LV cells were assessed by Western blot analysis. (H, I) The mRNA levels of PXR and MDR1 in GV358‐LV cells were quantified by Real‐time PCR.

Previous evidence has highlighted the synergistic effects of FLCWK in enhancing the inhibitory action of 5‐FU on colon tumour growth in AOM/DSS mice.^24^ Since the MDR1 gene expression induced by 5‐FU is primarily controlled by PXR, we delved into the impact of FLCWK on 5‐FU‐induced resistance in colon cancer cells with varying PXR expressions. In our cellular experiments, treatment with 5‐FU alone resulted in upregulation of PXR and P‐gp protein expressions in three cell lines (Figure 3A,D,G), accompanied by an increase in MDR1 mRNA expression as assessed by quantitative PCR (Figure 3C,F,I). Conversely, when cells were treated with both 5‐FU and FLCWK, PXR mRNA expression decreased in all cell types (Figure 3B,E,H), along with a reduction in P‐gp protein and MDR1 gene expression specifically in GV358‐LV cells.

### 3.6 FLCWK can potentiate 5‐FU to inhibit tumour size in xenotransplanted nude mice

**FIGURE 4:**
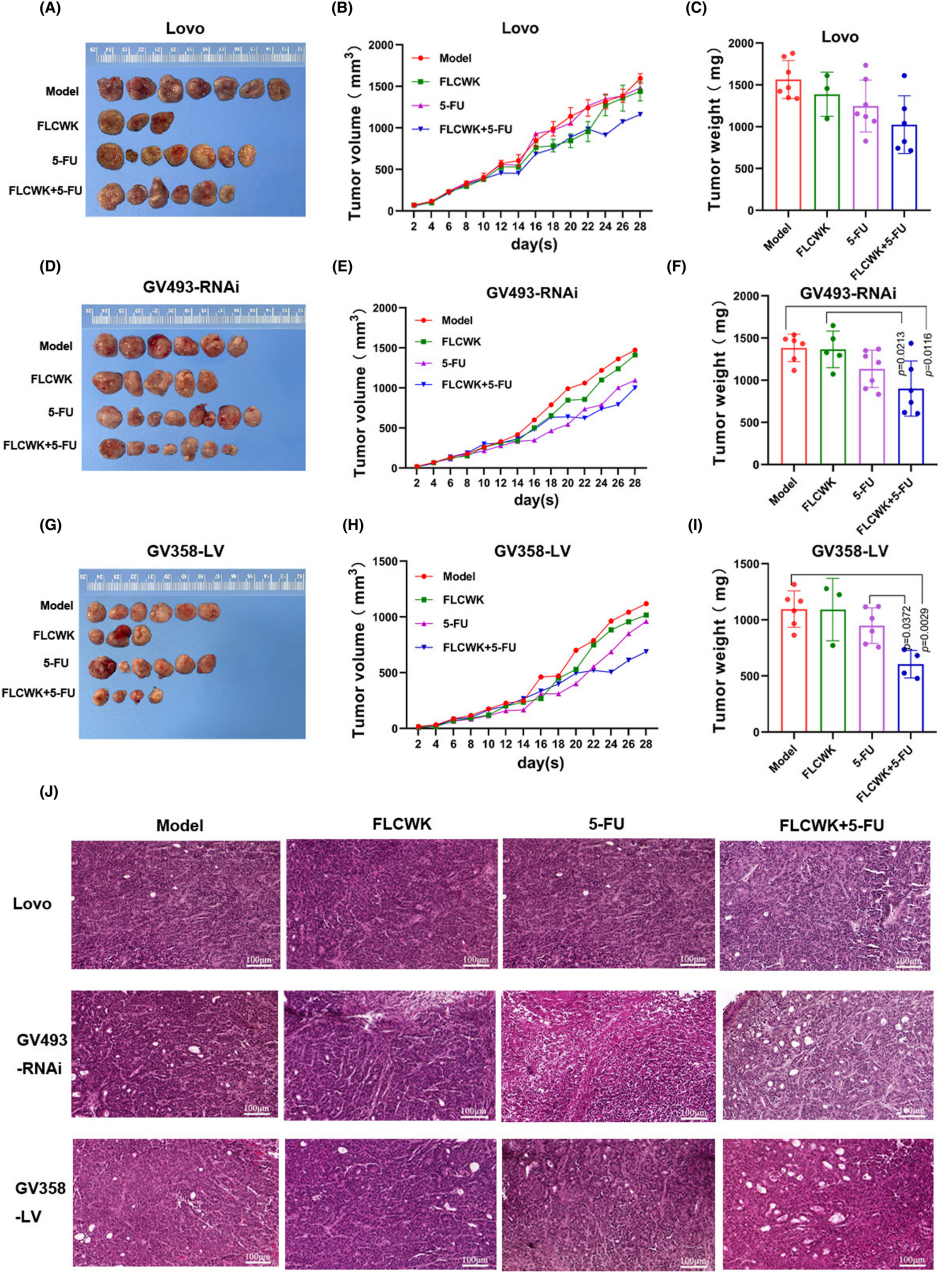
FLCWK combined with 5‐FU inhibits tumour growth in GV358‐LV cells xenotransplanted nude mice. (A, D, G) Tumour picture of Lovo cells, GV493‐RNAi cells and GV358‐LV cells xenotransplanted nude mice. (B, E, H) Tumour volume growth curves after administration of Lovo cells, GV493‐RNAi cells and GV358‐LV cells xenotransplanted nude mice. (C, F, I) Tumour weights of xenotransplanted nude mice. (J) HE staining of tumour tissue from xenotransplanted nude mice.

In a subcutaneous xenograft model in nude mice, tumours in mice overexpressing PXR exhibited significantly slower growth compared to those in Lovo cell xenotransplanted nude mice (Figure 4A,B,G,H). In the GV493‐RNAi cell xenotransplanted nude mice, initially, the tumours were smaller than those in the Lovo cell xenotransplanted nude mice, but later on, there was no significant difference in tumour size (Figure 4A,B,D,E). A noteworthy observation from this model was that individual administration of 5‐FU or FLCWK in all three cell lines did not lead to significant inhibition of tumour growth. However, simultaneous administration of both drugs notably enhanced tumour suppression. This enhancement was particularly striking in GV358‐LV cell xenotransplanted nude mice, where the combined treatment resulted in an inhibition rate of 47.04% compared to the control group (Figure 4C,F,I). These findings indicate that the combined efficacy of FLCWK and 5‐FU is maximized when PXR expression is elevated.

Through pathological tissue sectioning, we can observe an increase in the number, density, large nuclei and deep staining of tumour cells in three cell lines xenotransplanted nude mice model groups. After treatment with FLCWK combined with 5‐FU, it was observed that the nuclear staining became lighter, and different types of necrosis, nuclear condensation and reduced infiltration of inflammatory cells occurred in the tumour tissue. The GV358‐LV cell xenotransplanted nude mice had lighter nuclear staining and fewer nuclear divisions compared to the other two groups, with the combination therapy group showing the most significant changes within the overexpression group.(Figure 4J).

### 3.7 FLCWK can reverse the 5‐FU resistance induced by PXR activation and suppresses the IL‐6/STAT3 pathway in xenotransplanted nude mice models

**FIGURE 5:**
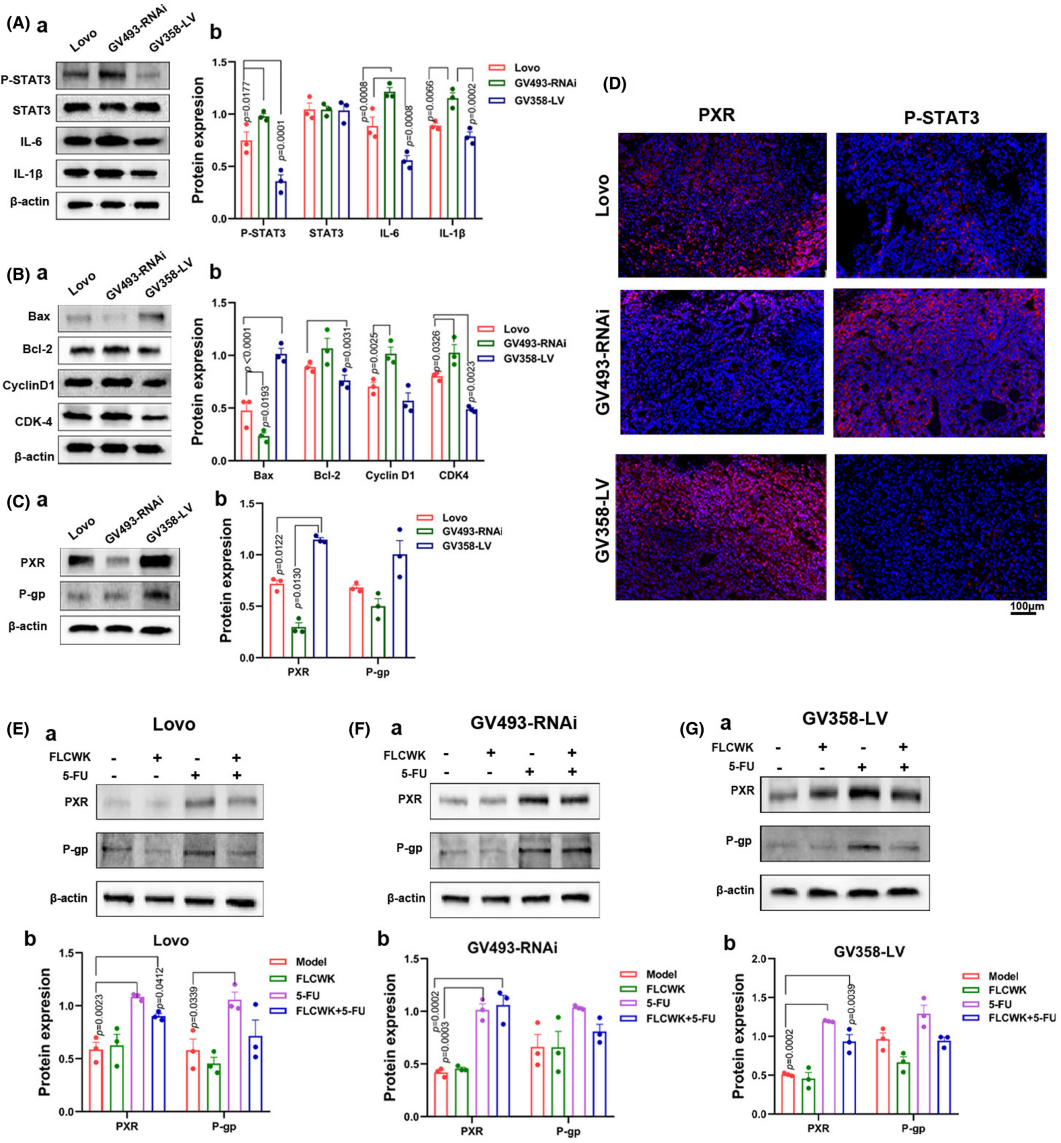
Overexpression of PXR can inhibit the IL‐6 pathway and promote the reversal of 5‐FU resistance by FLCWK in xenotransplanted nude mice. (A) The protein levels of P‐SATA3, STAT3, IL‐6 and IL‐1β in tumour tissues from xenotransplanted nude mice were assessed by Western blot analysis. (B) The protein levels of Bax, Bcl‐2, CyclinD1 and CDK‐4 in tumour tissues from xenotransplanted nude mice were assessed by Western blot analysis. (C) The protein levels of PXR and P‐gp in tumour tissues from xenotransplanted nude mice were assessed by Western blot analysis. (D) Localization and expression levels of PXR and P‐STAT3 in tumour tissues from the xenotransplanted nude mice were detected by immunofluorescence staining. (E, F, G) The protein levels of PXR and P‐gp in tumour tissues from xenotransplanted nude mice were assessed after FLCWK and 5‐FU treatment by Western blot analysis.

These cellular findings were further corroborated in xenotransplanted nude mice models. Analysis of tumour tissues from the PXR‐expressing mouse models exhibited a significant decrease in P‐STAT3 expression, reduced levels of IL‐6 and IL‐1β proteins in GV358‐LV cells xenotransplanted nude mice models, along with an increase in Bax expression and decreases in Bcl‐2, Cyclin D1 and CDK‐4 proteins. Conversely, IL‐6 and IL‐1β protein levels were elevated in the tumour tissues of GV493‐RNAi cell xenotransplanted nude mice models (Figure 5A,B,D).

In a murine xenograft model, treatment with 5‐FU alone led to elevated expression levels of PXR and P‐gp in the tumour tissues of mice across all cell models. However, co‐administration of FLCWK and 5‐FU led to a decrease in P‐gp expression in the tumour tissues of these models. Notably, treatment with FLCWK alone in mice bearing Lovo cells and GV358‐LV cells xenotransplanted nude mice models also resulted in reduced P‐gp expression (Figure 5C,E–G). These findings suggest that FLCWK may counteract the increase in P‐gp induced by 5‐FU, thereby reversing 5‐FU resistance in colon cancer.

### 3.8 FLCWK can potentiate 5‐FU to inhibit colon tumour growth in CAC Mice

**FIGURE 6:**
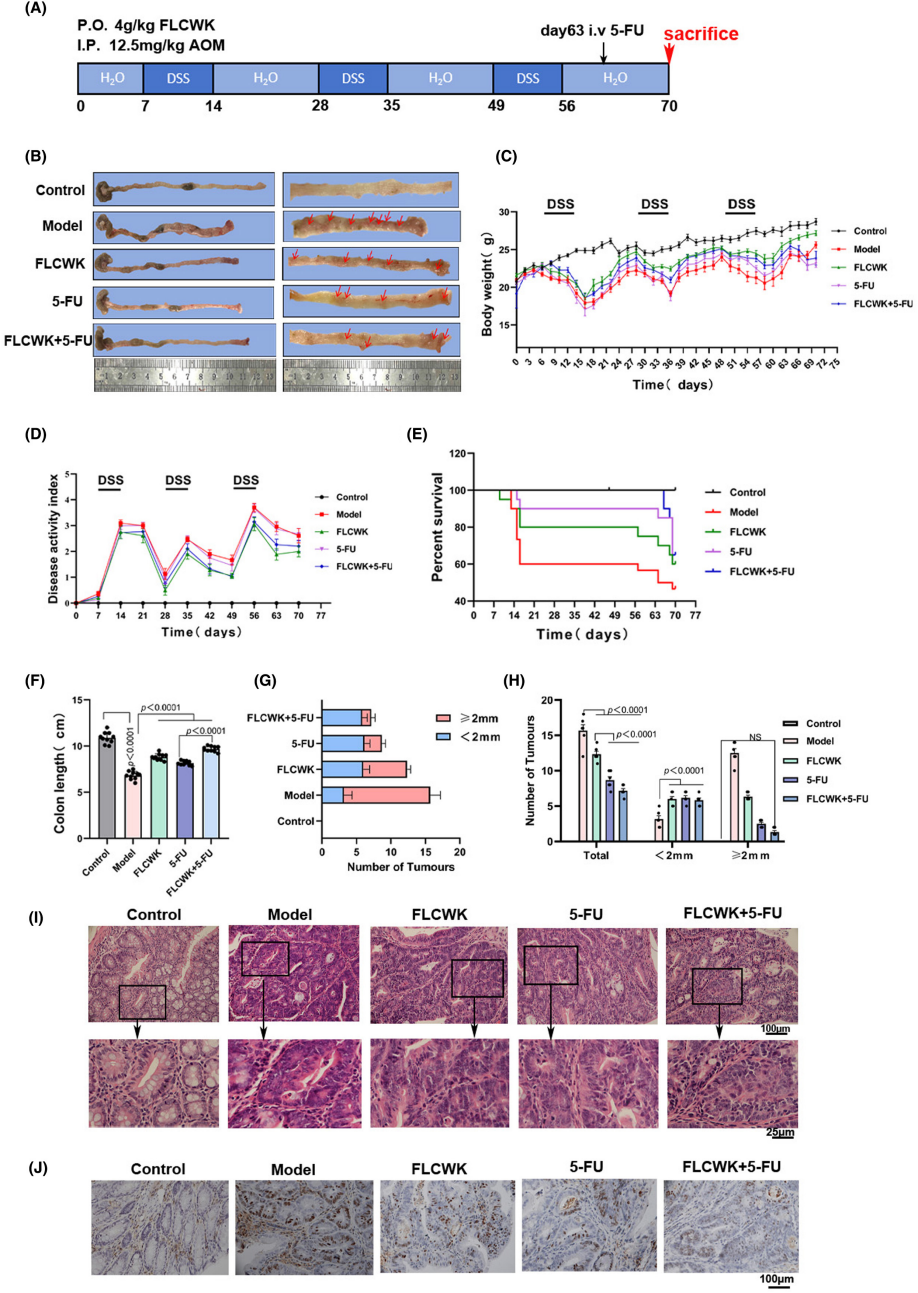
FLCWK can potentiate 5‐FU to inhibit the development of colitis‐associated colon cancer. (A) Establishment of AOM/DSS colon cancer mice model and method of drug administration. (B) Representative pictures of colon tissues in different groups on day 70. (C) Curves of changes in body weights of mice in different groups. (D) DAI of mice in the five groups. (E) Survival curves of mice in different groups. (F) Colon length of mice in different groups. (G, H) Number of tumours in different groups of mice. (I) HE staining of colonic tissues from different groups of mice. (J) Immunohistochemistry for Ki‐67 in colorectal tumours from the five groups of mice.

To further investigate the impact of FLCWK combined with 5‐FU on colon cancer, we conducted a study using a CAC mice models (Figure 6A). Post‐modelling observations revealed the presence of tumours of varying sizes covering the inner walls of the mice's colons, along with significant bleeding and a noticeable reduction in colon length (Figure 6B,F). During the third cycle of DSS modelling, there were varying mortality rates among the groups, with the highest survival rate recorded in the FLCWK+5‐FU group (Figure 6D,E), which also exhibited significantly fewer tumours on the inner colon walls compared to other groups (Figure 5C,G,H).

Histopathological examination uncovered the presence of multiple polyp‐like and nodular tumours on the colon surface, substantial thickening of the intestinal wall, prominent lymphocytic aggregation at the base, and alterations in crypt architecture characterized by enlarged nuclei. Following treatment, all groups showed improvements in inflammation, with the combined treatment group displaying significantly less inflammatory cell infiltration in the colon compared to the 5‐FU group and the FLCWK group (Figure 6I,J).

### 3.9 FLCWK reverses 5‐FU resistance by decreasing P‐gp expression to treat CAC mice

**FIGURE 7:**
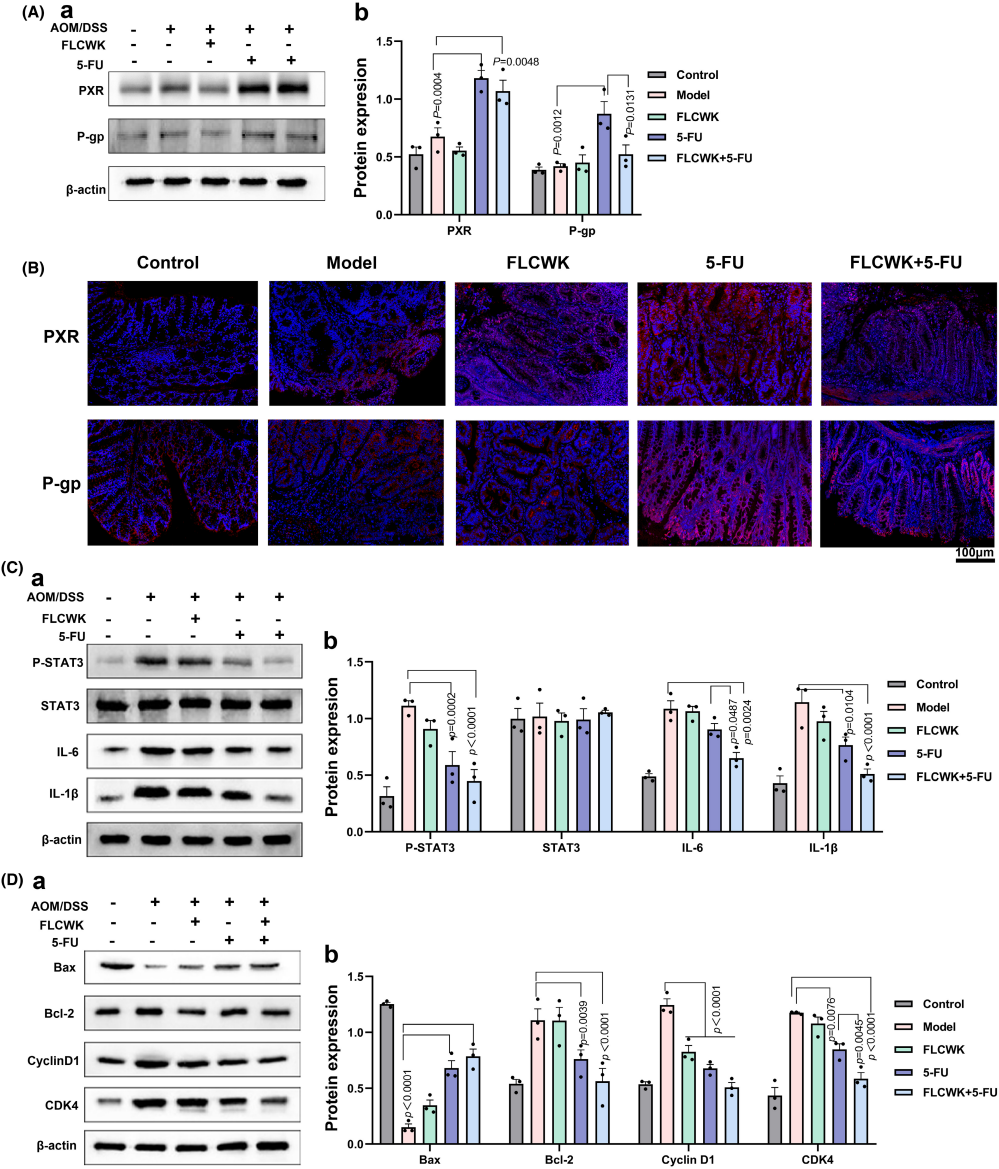
FLCWK potentiates 5‐FU inhibition of the IL‐6/STAT3 pathway while decreasing 5‐FU resistance in AOM/DSS colon cancer mice model. (A) The protein levels of PXR and P‐gp in different groups of mice colon tissues. (B) The localization and expression levels of PXR and P‐gp were determined in mice colon tissues by immunofluorescence staining. (C) The protein levels of P‐SATA3, STAT3, IL‐6 and IL‐1β in different groups of mice colon tissues. (D) The protein levels of Bax, Bcl‐2, CyclinD1 and CDK‐4 in different groups of mice colon tissues.

We further investigated the impact of FLCWK on 5‐FU resistance in a CAC mouse model. After inducing tumours with AOM/DSS (Figure 6A, 7A), an upregulation in PXR protein expression was observed. Treatment with 5‐FU alone notably increased PXR protein levels. However, concurrent administration of FLCWK and 5‐FU resulted in a decrease in PXR expression. P‐gp expression, influenced by various factors, did not show significant changes in the model group compared to the control, or in the FLCWK group compared to the model group. Yet, P‐gp expression in the 5‐FU group was significantly higher than in the model group, and notably lower in the FLCWK +5‐FU group compared to the 5‐FU group alone. Confocal microscopy analysis revealed that while 5‐FU activated P‐gp protein expression, the fluorescence intensity of PXR and P‐gp was prominently reduced in the FLCWK +5‐FU group (Figure 7B). These findings suggest that FLCWK can diminish P‐gp expression, thereby reversing 5‐FU resistance and providing a potential therapeutic approach for CAC.

### 3.10 FLCWK enhances the efficacy of 5‐FU in suppressing the IL‐6/STAT3 pathway in CAC treatment

Finally, we assessed the impact of FLCWK and 5‐FU on the IL‐6/STAT3 pathway. Protein analysis of colon tissues from the various groups revealed that the modelling induced activation of STAT3, leading to a significant increase in P‐STAT3, IL‐1β, IL‐6, Bcl‐2, Cyclin D1 and CDK‐4 levels, while Bax protein expression was decreased. Following treatment, the FLCWK +5‐FU group exhibited markedly reduced expression of P‐STAT3, Bcl‐2, Cyclin D1 and CDK‐4, along with elevated Bax protein levels compared to the other groups (Figure 7C,D). These results suggest that FLCWK can enhance the effectiveness of 5‐FU in suppressing the expression of the IL‐6/STAT3 pathway in CAC.

## 4 DISCUSSION

The primary aim of our study was to explore whether the combination of FLCWK could enhance the anticancer effects of the chemotherapeutic drug 5‐FU and counteract its resistance. The nuclear receptor superfamily, particularly PXR, plays a crucial role in maintaining intestinal homeostasis and regulating various pathophysiological processes. The regulatory influence of PXR on tumour progression involves a complex network of interactions.^28^, ^29^ Previous research has highlighted the presence of PXR in different cancer types, including breast, colon, liver, oesophageal and prostate cancers.^30^, ^31^, ^32^, ^33^ Specifically, in colon cancer research, PXR overactivation has been associated with increased tumour cell proliferation and inhibition of apoptosis.^34^ Our findings indicated that the combined treatment with FLCWK and 5‐FU, following PXR activation, effectively suppressed the proliferation of colon cancer cells and promoted apoptosis. This effect may be linked to the downregulation of proliferative proteins like Cyclin D1 and the upregulation of pro‐apoptotic proteins such as B cl‐2. In our experiments on nude mice, tumours in mice with high PXR expression were notably smaller than those in PXR‐silenced mice. Furthermore, the inhibitory effect on tumour growth was particularly pronounced in PXR‐activated mice treated with FLCWK in combination with 5‐FU.

While 5‐FU is a cornerstone of cancer therapy, the challenge of resistance in cancer cells poses a significant obstacle, often resulting in only a 20%–30% inhibition rate in colon cancer cells.^22^, ^35^ Researchers worldwide have been exploring various treatment approaches to enhance the anticancer efficacy of 5‐FU and address clinical resistance.^11^, ^21^, ^22^ One of the primary drivers of MDR in tumours is the overactivation of MDR1 in tumour cells, leading to increased expression of the transcriptional protein P‐gp. Our investigation demonstrated that 5‐FU could induce the upregulation of MDR1 through the activation of PXR, influencing treatment outcomes. However, the combination of FLCWK with 5‐FU resulted in the downregulation of MDR1, enhancing treatment effectiveness. Therefore, we propose that FLCWK may decrease MDR1 expression, thereby reversing the resistance induced by 5‐FU.

**FIGURE 8:**
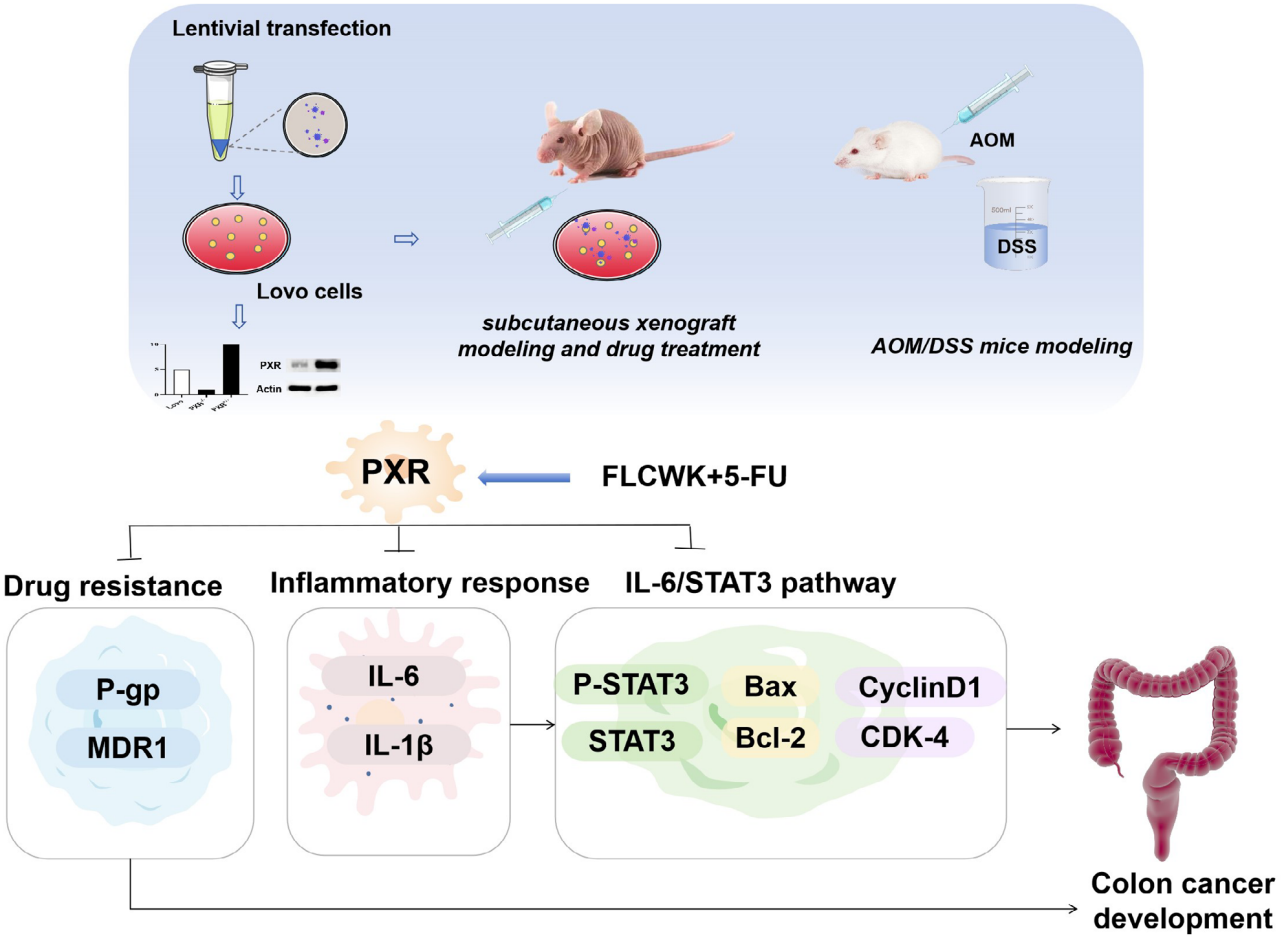
Graphical abstract. The potential therapeutic capabilities of FLCWK in managing colon cancer, particularly in conjunction with 5‐FU to bolster the inhibition of colon cancer initiation and progression.The IL‐6/STAT3 pathway can interact synergistically with the nuclear receptor PXR to modulate alterations in the tumor microenvironment and mitigate resistance mechanisms.

Numerous studies have underscored the pivotal role of the IL‐6/STAT3 pathway in the transition from colitis to colon cancer.^36^ It is well‐established that both STAT3 and interleukin‐6 IL‐6 are commonly found in the tumour microenvironment of human and murine colorectal cancer,^37^ correlating with reduced survival rates and increased recurrence risks.^38^ Prior research has indicated that PXR can regulate the IL‐6/STAT3 pathway, leading to the downregulation of associated transporters. In our investigation using the AOM/DSS colon cancer model, we explored the interplay between PXR and the IL‐6/STAT3 pathway. Our findings revealed that PXR was activated during the development of CAC, resulting in an increase in P‐gp levels. Concurrently, the IL‐6/STAT3 pathway was activated, accompanied by elevated expressions of downstream regulatory proteins (CyclinD1, CDK‐4 and Bcl‐2). Upon pharmacological intervention, treatment with 5‐FU alone effectively inhibited the IL‐6/STAT3 pathway but also led to elevated levels of both PXR and P‐gp. In contrast, combination therapy demonstrated that the addition of FLCWK enhanced the inhibitory effect of 5‐FU on the IL‐6/STAT3 pathway, resulting in decreased levels of PXR and P‐gp compared to 5‐FU alone. Additionally, reductions in PXR and P‐gp levels were observed in the FLCWK group. The inhibitory effect on P‐gp through this pathway overrode the activating effect of PXR, resulting in diminished P‐gp expression. In the nude mouse tumour model, PXR activation was associated with the inhibition of STAT3 phosphorylation and a decrease in tumour growth rate, exhibiting differences from the findings in the CAC mouse model. We propose that this disparity may arise from the prolonged progression of CAC, where PXR plays diverse roles in the transition from inflammatory bowel disease to cancer, significantly influencing colon cancer progression, metastasis, migration and chemotherapy resistance, while being closely linked to tissue specificity, inflammatory environment and individual variabilities.^39^, ^40^, ^41^, ^42^ In conclusion, our study underscores the therapeutic potential of FLCWK in the management of colon cancer, particularly when used in combination with 5‐FU to enhance the inhibition of colon cancer initiation and progression. Furthermore, the interaction between the IL‐6/STAT3 pathway and the nuclear receptor PXR can synergistically modulate changes in the tumour microenvironment and mitigate resistance mechanisms (Figure 8). Thus, the exploration of complementary agents that can enhance the efficacy of 5‐FU through the bidirectional IL‐6‐STAT3‐PXR pathway shows promise in improving the chemotherapeutic outcomes of 5‐FU against CAC, taking into account both pharmacodynamic and pharmacokinetic considerations.

## AUTHOR CONTRIBUTIONS

**Lifan Zhong:** Data curation (equal); funding acquisition (equal); writing – original draft (equal); writing – review and editing (equal). **Qianru Wang:** Data curation (equal); formal analysis (equal). **Zhixiong Kou:** Data curation (equal); formal analysis (equal). **Lianfang Gan:** Formal analysis (equal); methodology (equal). **Zhaoxin Yang:** Formal analysis (equal); methodology (equal). **Junhua Pan:** Methodology (equal). **Ling Huang:** Project administration (equal); resources (equal). **Yunqiang Chen:** Funding acquisition (equal).

## FUNDING INFORMATION

This work was supported by the Hainan Provincial Natural Science Foundation of China (grant number 821MS047, 2021), Hainan Medical University Cultivation Foundation of China (grant number HYPY2020030, 2020), the National Natural Science Foundation of China (grant number 81760674,2017), Hainan Province Clinical Medical Center (Department of TCM Rehabilitation of Sanya TCM Hospital).

## CONFLICT OF INTEREST STATEMENT

The authors have no conflicts of interest to declare.

## Data Availability

The data that support the findings of this study are available from the corresponding author upon reasonable request.
